# The Properties and Uses of Iodine

**Published:** 1855-09

**Authors:** W. G. Piper

**Affiliations:** Winterset, Iowa


### THE PROPERTIES AND USES OF IODINE WITH REPORT OF A CASE

BY W. G. PIPER, M. D., WINTERSET, IOWA.

IT is among the impertant duties which every Practitioner of Medicine owes to the profession, to contribute all the important facts and suggestions that may fall under his own observation. It is only by such a course that the profession can make that progression in the procuration of facts, and the development of principles which are to guide us in our daily conflicts with the protean forms of disease that afflict our race.

We live in an age of great mental improvement, at least, one that surpasses all others in activity, enterprise and innovation.—Among these evidences of intelligence and industry, the profession of medicine is far from being in the back ground. Within the last few years we have had several valuable accessions to our science, and have every reason to believe that the spirit of inquiry which led to these discoveries will not die out, when the present generation has passed away. My attention has for the past few years been directed to the medicinal properties of Iodine. It has been an agent that has long been known to exert a powerful and extensive influence on the animal economy, consequently, we cannot at this time refer to all the indications for its use. Perhaps, no country practitioner has used it more extensively than I, in my short career of practice; and my suggestions are based upon my experience in the use of the remedy. Iodine, in small doses, is a tonic, increases the appetite and improves faulty nutrition.

It also exerts a powerful influence upon the absorbents and glandular system. Being satisfied that it possessed this power in an eminent degree, I have been induced to test its virtues in the cure of phthisis pulmonalis. Many remedies have been suggested for the cure of this formidable malady, but all in turn have been abandoned forever, and the profession generally induced to believe that no remedy yet discovered, could be relied on in the treatment of this disease. Recently, however, there have been some eminent physicians who firmly believe that incipient Phthisis Pulmonalis may occasionally, in a certain stage, be cured. If it can be successfully treated in one case, or even a spontaneous cure be effected, have we not reason to hope for a similar result in many such cases. Should we be able to discover a remedy that will excite the absorbents to such a degree as to remove tuberculous matter, subdue the diathesis, and by so doing, prevent the deposition of other tubercles—then will we have arrived at an important period in the history of medicine; then will thousands of the most gifted and beautiful be saved from a premature grave.

As evidences of the curability of incipient phthisis pulmonalis, I desire to introduce a brief synopsis of one case that was successfully treated with Iodine.

Mr. P. aged 54 years on the 14th day of Nov., 1854, labored under the following symptoms: Pain in the breast, referred to the apex of the lung, more or less cough, particularly when lying down, and in the morning. Expectoration of a tough mucus consistence, and but little thrown up. Fever, with regular exacerbations, followed with night sweats, gradual emaciation without sufficient visible cause, appetite capricious, patient had for some days been troubled with diarrhœa. The digestive organs did not perform their healthy functions. He was very ingenious in presenting arguments to convince me that his lungs were not seriously affected. He was able to be up part of the day, but could not endure much exercise. The physical signs were such in connection with the rational evidences in the case, as to induce me to believe that it was an unmistakable case of incipient phthisis pulmonalis. At the apex and the left lobe of the lungs, there was dullness on percussion. This condition was not found to exist in any other part of the lungs. The breathing was insufficient and coarse, there was a peculiar resonance of the voice, and abnormal click-like sound. When he spoke or coughed there was produced a kind of sound more easily imagined than described. Broncophon and bronchial respiration could be heard, and differed from that of health, by being distinctly audible near the humoral extremity of the clavicle. With such an array of symptoms, there seemed but little grounds for doubting the existence of tubercular deposits.—But still the evidence was not absolutely certain, yet the symptoms could not be distinguished from those which have characterized incipient phthisis pulmonalis, which in hundreds of cases have not been interrupted, but passed on through the different stages of the disease to a fatal termination.

Without making known to him or his family the character of the disease, I directed Pil. Hyd. grs. vi; Opi. gr. i; to be taken at bedtime.

In a few days I directed the following mixture:

℞ Compound Syrup Sarsaparilla, oz. iii.
Tinct. Iodine, drs. iii.

A teaspoonful to be taken every six hours. I also directed the Nitro-Muriatic bath four times per week. He was also directed to use a nutritious diet, in moderate quantities. He continued to use the Iodine, without interruption, for three months, during which time I occasionally used a Dover's powder or a dose of morphine to procure rest. Several weeks passed without any material change in the symptoms, but rather an aggravation. The expectoration became puriform, streaked occasionally with blood. His feet became œdematous.

Notwithstanding all this, the remedy was continued, and part of the time given in larger doses. About the first of February, 1855, the unfavorable symptoms began gradually to disappear, and about the 15th day of said month, which was three months after he commenced the use of the Iodine, the expectoration and the cough ceased, and he was so much improved in every respect, that the remedy was discontinued, and he is now a healthy man for his age without any perceivable symptoms of Phthisis Pulmonalis.

I do not wish to be understood as pronouncing all cases of phthisis pulmonalis curable; facts will not justify such a conclusion. But that the disease has been frequently cured, I do not entertain a doubt. The first and most important in the treatment of this disease, is to improve faulty nutrition. Cod liver oil has gained much reputation in the cure of phthisis. But I incline to believe that the virtue of this remedy may be attributed to this fact that it is easily assimilated, consequently its remedial effects are referable more to its nutritious than to its medicinal properties.—We are warranted in drawing the conclusion, that if, during the advance of phthisis pulmonalis, those means can be discovered which will keep up the strength and nutritive functions of the economy, such tubercular exudations as have occurred may be absorbed, and that even large ulcerations will heal up and cicatrize. The most important point, practically, is to ascertain what these means are, and how they may be put into operation. I am not one to believe that phthisis pulmonalis is, in all cases necessarily fatal.—With such testimony before us, however, I hope no physician will be so skeptical in regard to the cure of phthisis pulmonalis as to reject all curative treatment. If the disease is curable, the physician owes it to humanity not only to seek out a remedy, but to persevere in its use, until he is satisfied that a longer persistence will result in no good, and particularly where remedies shall have proved a source of annoyance rather than profit.

(We would respectfully suggest whether the Iodine is not in these cases, rather an *analeptic* than tonic. We are much gratified that he has reported the above case, as it will encourage others to further perseverence. Science, in its humane offices, *must* and *will* yet discover some agent which will not only prevent exudation, but will induce its absorbtion and ultimate depuration. The great progress already made, augurs favorably for its capacity, and the future accomplishment of so great an object yet we dare not hope to enjoy the life time to witness it, still we believe that period is not far distant. All should contribute by their efforts to the work of discovery, and place the results of their labors before their brethern. One improvement has been made, and that is the abandonment of antiphlogistics and the use of mercurials, as well as depressing expectorants. ED.)

